# Formulating a Horseradish Extract in Phospholipid Vesicles to Target the Skin

**DOI:** 10.3390/pharmaceutics16121507

**Published:** 2024-11-23

**Authors:** Maria De Luca, Luca Casula, Carlo Ignazio Giovanni Tuberoso, Ramon Pons, Maria del Carmen Morán, María Teresa García, Giuseppe Martelli, Antonio Vassallo, Carla Caddeo

**Affiliations:** 1 Department of Science, University of Basilicata, Viale dell’Ateneo Lucano 10, 85100 Potenza, Italy; 2 KAMABIO Srl, Via Al Boschetto 4/B, 39100 Bolzano, Italy; 3 Department of Life and Environmental Sciences, University of Cagliari, S.P. Monserrato-Sestu km 0.700, 09042 Cagliari, Italy; luca.casula@unica.it (L.C.); caddeoc@unica.it (C.C.); 4 Department of Surfactants and Nanobiotechnology, Institute for Advanced Chemistry of Catalonia (IQAC-CSIC), c/Jordi Girona, 18-26, 08034 Barcelona, Spain; ramon.pons@iqac.csic.es (R.P.); teresa.garcia@iqac.csic.es (M.T.G.); 5 Department of Biochemistry and Physiology, Physiology Division, Faculty of Pharmacy and Food Science, University of Barcelona, Avda. Joan XXIII 27-31, 08028 Barcelona, Spain; mcmoranb@ub.edu; 6 Institute of Nanoscience and Nanotechnology—IN2UB, University of Barcelona, Avda. Diagonal 645, 08028 Barcelona, Spain; 7 Department of Basic and Applied Sciences, University of Basilicata, Viale dell’Ateneo Lucano 10, 85100 Potenza, Italy; giuseppe.martelli@unibas.it; 8 Department of Health Sciences, University of Basilicata, Viale dell’Ateneo Lucano 10, 85100 Potenza, Italy; 9 Spinoff TNcKILLERS Srl, Viale dell’Ateneo Lucano 10, 85100 Potenza, Italy

**Keywords:** horseradish root, extract, nanovesicles, skin delivery, cytocompatibility, antioxidant

## Abstract

**Background/Objectives**: Horseradish (*Armoracia rusticana* L.) roots—largely used in traditional medicine for their multiple therapeutic effects—are a rich source of health-promoting phytochemicals. However, their efficacy can be compromised by low chemical stability and poor bioavailability. Incorporation into phospholipid vesicles is often proposed to tackle this problem. **Methods**: In this study, a hydroalcoholic extract was produced from horseradish roots. The extract was characterized by UPLC-MS and HPLC-PDA and formulated in conventional liposomes and Penetration Enhancer-containing Vesicles (PEVs) for skin application. **Results**: The obtained nanovesicles were small in size (<100 nm), negatively charged, uni/bilamellar, and with high values of entrapment efficiency (>85%) for the flavonoids identified in the extract. Both the free and the nanoformulated extract showed optimal biocompatibility, measured as the absence of hemolysis of erythrocytes and absence of cytotoxicity in skin cell lines. Furthermore, the nanoformulations displayed antioxidant activity in vitro. **Conclusions**: The proposed nananoformulations could be exploited to counteract oxidative stress involved in the pathogenesis and progression of numerous skin disorders.

## 1. Introduction

*Armoracia rusticana* L., commonly known as horseradish, is a perennial plant of the Brassicaceae family [1]. Originally from eastern Europe, it is nowadays grown worldwide and used as a spice for the hot, pungent flavor of its roots [2]. Horseradish is a source of phytochemicals with health-promoting properties, such as glucosinolates (sinigrin, glucobrassicin, neoglucobrassicin, and gluconasturin), which are responsible for the characteristic flavor and aroma [3,4]. The roots are also used in traditional medicine for their multiple therapeutic effects, as a remedy for headache or rheumatic pain, cough, bronchitis, and other respiratory conditions [5]. Among the numerous compounds contained in horseradish root extracts, the ones characterized by a medium polarity—e.g., fatty acids like linoleic acids—are responsible for the antiphlogistic activity by blocking the COX and 5-LOX pathway [6]. Horseradish roots also contain ascorbic acid, which has been reported to strengthen the body’s defenses [7], and flavonoids, particularly kaempferol and quercetin, which have been demonstrated to provide cancer chemoprevention by protecting human lymphocytes from DNA damage [4]. In light of these biological activities, numerous studies have investigated the extraction of the main bioactive compounds of horseradish, such as glucosinolates, isothiocyanates, and organo-sulfur compounds. However, some issues concerning their chemical stability and bioavailability have been highlighted [8,9]. Most plant-derived products are characterized by poor aqueous solubility and low permeability, resulting in limited absorption in vivo. To overcome these issues, nanoparticulate delivery systems have been proposed for their remarkable advantages over traditional systems [10,11]. Among the nanocarriers explored, phospholipid-based vesicles (e.g., liposomes) have proven to be a promising and attractive strategy to entrap whole-plant extracts or isolated phytoconstituents [12].

Being biocompatible and adaptable, phospholipid-based vesicles can entrap a variety of compounds, providing protection from degradation, increasing their solubility, modulating their release, and facilitating their absorption through biological membranes [13]. With a specific focus on dermal and transdermal delivery, the scientific community have paid particular attention to the preparation of phospholipid vesicles with chemical penetration enhancers (e.g., surfactants, ethanol), such as PEVs (Penetration Enhancer-containing Vesicles), transfersomes, and ethosomes, that can increase the penetration of their cargo through the skin by interacting with its components [14,15].

In this study, an extract from *Armoracia rusticana* L. roots was firstly analyzed to identify the predominant components and secondly incorporated in phospholipid-based vesicles, namely conventional liposomes, and liposomes modified with ethanol—Et-PEVs [16]. The vesicles’ size, homogeneity, surface charge, lamellarity, and antioxidant activity were determined. Moreover, the biocompatibility of the nanoformulations was assessed by evaluating the possible cytotoxicity in different skin cell lines (i.e., fibroblasts and keratinocytes) and the hemolytic activity in rabbit erythrocytes.

## 2. Materials and Methods

### 2.1. Materials

Standards of uridine, tyrosine, phenylalanine, tryptophan, vanillic acid, tyrosol, sinigrin, glucobrassicin, and kaempferol-3-*O*-rutinoside were from TransMIT (Giessen, Germany) and Extrasynthese (Genay Cedex, France). Lipoid S75 (soy phospholipids with 70% phosphatidylcholine; S75) was from Lipoid GmbH (Ludwigshafen, Germany). Ethanol 96% was from Sigma/Merck (Milan, Italy). The materials used for cell experiments were from Lonza (Verviers, Belgium). Murine Swiss albino fibroblasts (3T3), immortal human keratinocytes (HaCaT), and squamous carcinoma cells (A431) were provided by Celltec UB (Barcelona, Spain).

### 2.2. Harvest and Extraction

Horseradish (*Armoracia rusticana* L.) roots were harvested in Oliveto Lucano (Matera, Italy), cleaned, cut into pieces, left to dry for 72 h, and ground into a fine powder. The powder was dispersed in ethanol 70% (1:2, *w*/*v*), sonicated for 30 min, and macerated for 24 h at room temperature, according to a procedure previously described [17]. The macerate was concentrated, and the resulting solid extract was stored at 4 °C.

### 2.3. UPLC/MS and HPLC-PDA Analyses

The horseradish extract was analyzed by a UPLC/MS system according to De Luca et al. [18], using a 1290 Infinity II UPLC equipped with a 6560 ion mobility-QToF with the Electrospray Ionisation (ESI) source in negative ion mode (Agilent Technologies Inc., Palo Alto, CA, USA). A MassHunter Workstation Acquisition software v. B.09.00 (Agilent Technologies) was employed for data acquisition and processing; a MassHunter Workstation Qualitative Analysis software v. 10.0 (Agilent Technologies) was employed for ESI/QToF MS data analysis; MassHunter METLIN metabolite PCDL database v. B.08.00 (Agilent Technologies), and Sirius^®^ software v. 5.8.2 were employed for tentative identification of metabolites, prediction of fragmentation, and molecular formulae [19,20].

Target compounds were quantified by an HPLC-PDA detection method described by De Luca et al. [21] using a 1260 Infinity II HPLC system with a G4212B photodiode array detector (Agilent Technologies). The horseradish extract was dissolved in an 80:20 *v*/*v* methanol/water mixture (1:50 *w*/*v* extract/solvent ratio) and diluted 1:1 *v*/*v* with 0.22 M phosphoric acid prior to injection. Detection and quantification were performed at characteristic wavelengths: 360 nm for flavonols, 280 nm for hydroxybenzoic acids, and 210 nm for the other compounds. An OpenLab data system v. 2.51 (Agilent Technologies) was employed for chromatograms and spectra elaboration.

### 2.4. Horseradish Extract Formulations

Conventional liposomes and Et-PEVs were prepared as follows: for liposomes, the phospholipids (S75) and the horseradish extract (amounts are reported in Table 1) were dispersed in ultrapure water and sonicated with an ultrasound disintegrator (10 cycles of 5 s on/2 s off + 6 cycles of 2 s on/2 s off); for Et-PEVs, the phospholipids and the extract were dispersed in water and ethanol (amounts are reported in Table 1) and sonicated according to the same protocol used for liposomes.

The morphology of the vesicles was studied by means of cryogenic Transmission Electron Microscopy (cryo-TEM). The vesicle dispersions were observed under a JEM-2011 TEM (JEOL USA Inc., Peabody, MA, USA). Four milliliters were placed on a grid and vitrified into ethane using an EM GP cryo-preparation chamber (Leica Microsystems Inc., Deerfield, IL, USA). The TEM observation was carried out at an accelerating voltage of 200 kV.

The average diameter, polydispersity index, and zeta potential of the vesicles were measured by dynamic and electrophoretic light scattering techniques using a Zetasizer nano-ZS (Malvern Panalytical, Worcestershire, UK). The vesicle dispersions were diluted (1:100 *v*/*v*) with water prior to analysis at 25 °C.

In order to determine the entrapment efficiency of the liposomes and Et-PEVs, the horseradish extract nanoformulations were dialyzed against water [17]. Both non-dialyzed and dialyzed vesicle dispersions were diluted (1:100 *v*/*v*) with methanol and injected into HPLC with a PDA detector. Target compounds were quantified as described in Section 2.3.

A further characterization of the vesicles’ structure was gathered by Small-Angle X-ray Scattering (SAXS) analyses. The equipment and the experimental conditions used were described by De Luca et al. [18]. The SAXS curves were recorded every 20 min for 2 h to monitor sample stability, summed (with background subtraction), and fitted using an in-house procedure based on a Gaussian description of bilayers and a Levenberg–Marquardt minimization scheme [22].

### 2.5. Evaluation of Cytocompatibility and Antioxidant Activity

The biocompatibility of horseradish extract nanoformulations was evaluated as the absence of hemolysis in erythrocytes ex vivo and the absence of cytotoxicity in fibroblasts and keratinocytes in vitro. The antioxidant activity was estimated via DPPH (2,2-diphenyl-1-picrylhydrazyl) and FRAP (ferric reducing antioxidant power) assays.

#### 2.5.1. Hemolytic Activity

Erythrocytes isolated from rabbit blood were washed with Phosphate-Buffered Saline (PBS, pH 7.4) and suspended in PBS (10^9^ cells/mL). The hemolytic activity was assayed according to Perez et al. [23]. Then, 1 and 2 mg/mL of the horseradish extract solution (70:30 *v*/*v* ethanol/water) or the liposomes and Et-PEVs were tested. The samples were incubated, under stirring, with the erythrocytes for 10 min at room temperature and then centrifuged. Hemolysis was determined by reading the absorbance at 575 nm of the supernatant of the samples vs. that of the controls (i.e., erythrocytes in water—100% hemolysis—and erythrocytes in PBS—0% hemolysis).

#### 2.5.2. Cytotoxic Activity

3T3, HaCaT, and A431 cells were cultured in DMEM with glucose, fetal bovine serum, *L*-glutamine, penicillin, and streptomycin at 37 °C and 5% CO_2_. The 3T3 and HaCaT cells were seeded into 96-well plates at a density of 1 × 10^5^ cells/mL. The A431 cells were seeded at a density of 5 × 10^4^ cells/mL. All the cells were treated with a horseradish extract solution (70:30 *v*/*v* ethanol/water), or the nanoformulations were properly diluted with the culture medium to reach the required concentrations (0.1–20 mg/mL). After 24 h, the MTT (5 mg/mL in PBS) was added to the cells. After 3 h, dimethylsulfoxide was used to dissolve the formazan that formed due to the mitochondrial activity of viable cells. The absorbance was recorded at 550 nm. The cytotoxic activity was expressed as the percentage of viability of treated cells vs. untreated cells (control, 100% viability).

#### 2.5.3. Antioxidant Activity

For the DPPH assay, 40 μL of a horseradish extract solution (70:30 *v*/*v* ethanol/water) or the nanoformulations were added to a DPPH methanolic solution (25 mM) and incubated (in the dark, room temperature) for 30 min. The discoloration of the DPPH solution, corresponding to a decrease in absorbance, was recorded at 517 nm. The antioxidant activity (AA) was calculated as the percentage of the absorbance of the samples vs. that of the DPPH solution. The results were also expressed as Trolox equivalents (μg TE/mL solution) calculated by using a calibration curve (Trolox concentration range: 0–200 μg/mL).

For the FRAP assay, 40 μL of a horseradish extract solution (70:30 *v*/*v* ethanol/water) or the nanoformulations were added to the FRAP reagent and incubated (in the dark, room temperature) for 4 min. The development of a blue color, corresponding to an increase in absorbance, was recorded at 593 nm. The results were expressed as ferrous equivalents (μg FE/mL solution) calculated by using a calibration curve (ferrous sulfate concentration range: 13.9–1737.5 μg/mL).

### 2.6. Statistical Analysis

Student’s *t*-test was applied to determine significant differences between groups. For cell studies, the ANOVA test was applied to determine significant differences between data sets, and the Scheffé post-hoc test was applied for multiple comparisons. *p* values < 0.05 were considered significant.

## 3. Results

### 3.1. Quali-Quantitative Characterization of Horseradish Extract

**Figure 1:**
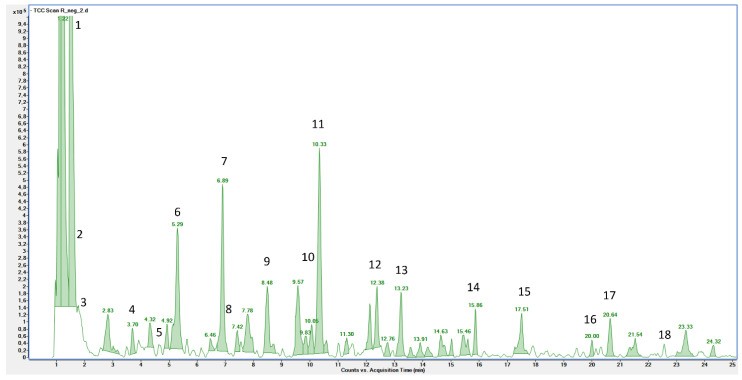
LC-MS profile of the horseradish extract. Peak identification is given in Table 2.

The horseradish extract was qualitatively analyzed by UPLC-MS, and target phenolic compounds were quantified by HPLC-PDA. Figure 1 reports the LC-MS profile of the extract. Its components were identified by comparing the *m*/*z* values and the experimental MS/MS spectra with data reported in the literature and in public repositories [19,20,24].

The identified compounds are listed in Table 2 according to their retention times, chemical formulae derived by mass measurement, MS/MS results, with the references used for identification, and the identification confidence levels [25]. Eighteen compounds were identified or tentatively identified, represented mainly by glucosinolates, hydroxybenzoic acids, flavonols, and amino acids.

**Table 2:** Compounds identified in the horseradish extract by LC-MS.

No.	Rt (min)	Identity	[M − H]^−^ * m/*z*	Molecular Formula	Δ (ppm)	MS/MS ^§^ *m*/*z*	References	Level #
1	1.56	2-Hydroxypropyl glucosinolate (Sinigrin)	358.0276	C_10_H_17_NO_9_S_2_	0.4033	259.0099 (7)/96.9601(100)/74.9907(56)	[26,27]	1
2	1.65	Uridine	243.0620	C_9_H_12_N_2_O_6_	−0.2597	152.0351(21)/110.0247(100)/82.0296(27)	[28]	1
3	1.78	Tyrosine	180.0668	C_9_H_11_NO_3_	0.1832	163.0402(100)/119.0505(87)	[29]	1
4	3.70	Phenylalanine	164.0715	C_9_H_11_NO_2_	−0.2021	147.0440(75)/103.0537(100)/72.0090(97)	[26]	1
5	4.62	Butyl glucosinolate and/or methylpropyl glucosinolate (glucocochlearine and/or glucoconringianine)	374.0587	C_11_H_21_NO_9_S_2_	0.2032	96.9605(67)/95.9633(100)/74.9913(55)	[26,27]	2
6	5.29	Dihydroxybenzoic acid hexoside I	315.0726	C_13_H_16_O_9_	0.4444	153.0189(57)/152.0119(100)/109.0294(45)	[30]	2
7	6.89	Dihydroxybenzoic acid hexoside II	315.0732	C_13_H_16_O_9_	0.6605	153.0193(100)/152.0116(27)/109.0298(80)	[30]	2
8	6.91	Tryptophan	203.0824	C_11_H_12_N_2_O_2_	−0.1732	-	[26]	1
9	8.48	3-Indolylmethyl glucosinolate (glucobrassicin)	447.0542	C_16_H_20_N_2_O_9_S_2_	0.4542	274.9973(5)/96.9604(100)/74.9906(9)	[6,26]	1
10	10.31	Dihydroxybenzoic acid	153.0191	C_7_H_6_O_4_	−0.2322	109.0295(100)	[31]	2
11	10.33	2-Phenethyl glucosinolate (gluconasturtiin)	422.0599	C_15_H_21_NO_9_S_2_	−0.9568	96.9608(100)/95.9520(45)/74.9907(24)	[6,26,27]	2
12	12.38	4-Methoxyglucobrassicin and/or Neoglucobrassicin	477.0646	C_17_H_22_N_2_O_10_S_2_	−0.1320	96.9608(100)/95.9520(49)/74.9910(36)	[6,26,27]	2
13	13.23	Vanilloyl exoside	329.0902	C_14_H_18_O_9_	1.4328	240.9989(7)	[32]	3
14	15.86	Phenethyl rutinoside	475.1821 [FA]	C_20_H_30_O_10_	0.2135	205.0701(19)/101.0229(21)/59.0143(100)	[33]	3
15	17.51	Sinapinic acid	223.0250	C_11_H_12_O_5_	−0.2823	179.0347(15) /109.0293(100)	[8,26]	2
16	20.00	Kaempferol-3-*O*-rutinoside	579.1354	C_26_H_28_O_15_	−0.1437	285.0408(71)/284.0323(100)	[6,31]	1
17	20.64	azelaic acid	187.0977	C_9_H_16_O_4_	−0.6825	125.0975(100)	[32]	2
18	22.53	Kaempferol di-pentoside	549.1251	C_25_H_26_O_14_	0.1209	399.0721(11)/285.0415(31)/284.0324(100)	[26,29]	2

* FA: formic acid adduct; ^§^ relative intensity is reported in brackets; # identification confidence level according to Blaženović et al. [25].

Five peaks were identified as glucosinolate derivatives, a class of sulfur- and nitrogen-containing compounds that characterize *A. rusticana* roots [9,26,27]. These compounds were characterized by the fragment [M−H]^−^ at *m*/*z* 96.96 corresponding to the [HSO_4_]^−^ ion [34]. They were attributed to 2-hydroxypropyl glucosinolate (**1**, sinigrin), butyl glucosinolate and/or methylpropyl glucosinolate (**5**, glucocochlearine and/or glucoconringianine,), 3-indolylmethyl glucosinolate (**9**, glucobrassicin), 2-phenethyl glucosinolate (**11**, gluconasturtiin), and 4-methoxyglucobrassicin and/or neoglucobrassicin (**12**). The amount of the four glucosinolates quantified by HPLC-PDA was 0.44 ± 0.03 mg/g dm, with 2-hydroxypropyl glucosinolate (sinigrin) being the most abundant (0.31 ± 0.0.2 mg/g dm, Table 3). Four peaks were characterized as nitrogen-containing compounds. Peak **2** with [M−H]^−^ at *m*/*z* 243.0620 was attributed to uridine, a ribonucleoside composed of uracil and riboside. The MS/MS spectrum was characterized by a fragment with [M−H]^−^ at *m*/*z* 110.0247 corresponding to a molecular formula C_5_H_5_NO_2_ and by the fragments [M−H]^−^ at *m*/*z* 152.0351 and at *m*/*z* 82.0296 [28]. This compound was confirmed by a pure standard and was never reported for *A. rusticana*. The other three compounds containing nitrogen were attributed to amino acids, namely tyrosine (**3**), phenylalanine (**4**), and tryptophan (**8**). They were already found in *A. rusticana* [26,29] and were confirmed by pure standards. The most abundant compound was phenylalanine (0.65 ± 0.07 mg/g dm), followed by tyrosine, uridine, and tryptophan.

Six hydroxybenzoic acid phenyl derivatives were identified. Three of them were attributed to dihydroxybenzoic derivatives due to the typical pseudomolecular ion [M−H]^−^ at *m*/*z* 153, which was associated with the dihydroxybenzoic unit [30]. Peaks **6** and **7**, with [M−H]^−^ at *m*/*z* 315, were attributed to dihydroxybenzoic acid hexosides [31]. Peak **10**, with [M−H]^−^ at *m*/*z* 153.02935, was attributed to dihydroxybenzoic acid due to the typical pseudomolecular ion [M−H]^−^ at *m*/*z* 109 [30,31]. Peak **15**, with [M−H]^−^ at *m*/*z* 223.0250 and pseudomolecular ion [M−H]^−^ at *m*/*z* 109, was attributed to a hydroxybenzoic acid derivative [35], namely sinapinic acid [8,26]. Peak **13**, with [M−H]^−^ at *m*/*z* 329.0902, was tentatively attributed to vanilloyl exoside [32]. Compound **14** presented a [M−H]^−^ at *m*/*z* 475.1821 corresponding to the formate-adduct of a molecule with a molecular weight of 430.1839 and a molecular formula of C_20_H_30_O_10_ that was tentatively attributed to phenethyl rutinoside [33]. This finding is consistent with the detection of phenethyl derivatives in aqueous extract of horseradish roots by GC-MS [6] and the presence of rutinosides detected by LC-MS. The two dihydroxybenzoic acid glucosides and the dihydroxybenzoic acid were the most abundant compounds (**6**, **7**, and **10**, respectively), and their sum (1.06 ± 0.08 mg/g dm; Table 3) accounted for 93% of the hydroxybenzoic acid phenyl derivatives.

Two peaks were attributed to flavonoid derivatives, namely kaempferol diglicosides, due to the typical pseudomolecular ion [M−H]^−^ at *m*/*z* 284. Peak **16**, with [M−H]^−^ at *m*/*z* 579.1354, was attributed to a kaempferol pentosyl exoside [6,26] and, after comparison with a pure standard, to kaempferol-3-*O*-rutinoside. Peak **18**, with [M−H]^−^ at *m*/*z* 549.1251, was attributed to a kaempferol dipentoside [26,29,31]. The total amount of flavonoids (Table 3) was 0.16 ± 0.01 mg/g dm, with kaempferol-3-*O*-rutinoside accounting for 0.11 ± 0.01 mg/g dm.

Peak **15**, with [M−H]^−^ at *m*/*z* 187.0977 and pseudomolecular ion [M−H]^−^ at *m*/*z* 125, was attributed to azelaic acid [32]. This dicarboxylic acid, which can be found in different vegetables, shows antimicrobial and antioxidant activities and can be used as a topical remedy to treat skin disorders [36].

### 3.2. Characterization of the Vesicles

The light scattering data showed that the empty liposomes were approximately 96 nm, slightly polydispersed (0.34), and highly negatively charged (−69 mV) (Table 4). In agreement with the observations found in the literature [37,38], the addition of ethanol leads to a significant decrease in the mean diameter (68 nm) of the empty Et-PEVs, which were more polydispersed (0.51) than empty liposomes but maintained a similar zeta potential value (−73 mV). The presence of ethanol in vesicle formulations is well known to increase the skin permeation of active agents due to an increase in the fluidity of the cell membranes’ lipids in dermal layers [39,40]. The loading of the horseradish extract had the opposite effect on liposomes and Et-PEVs: the former became smaller (from 96 nm to 84 nm), while the latter became larger (from 68 nm to 96 nm). Furthermore, the extract induced a reduction in the zeta potential values (from −69 mV to −52 mV for liposomes and from −73 mV to −63 mV for Et-PEVs; Table 4). Nevertheless, the charge was still highly negative to ensure electrostatic repulsion between vesicles and prevent aggregation [41]. A marked reduction in the polydispersity index of both systems (from 0.34 and 0.51 to ca. 0.22; Table 4) was also induced by the extract. This result points to a positive effect of the extract on the homogeneity of the nanoformulations. The only study in the literature on phospholipid vesicles loaded with an *A. rusticana* extract is reported by Pavaloiu et al., who prepared conventional liposomes using the thin-film hydration method followed by sonication and extrusion. The resulting liposomes had an average diameter of approximately 140 nm and a polydispersity index > 0.3. The entrapment efficiency—assessed using rutin as a standard—showed values ranging from 72 to 79% [42].

In the present study, two flavonoids, kaempferol-3-*O*-rutinoside and kaempferol di-pentoside, were identified in the horseradish extract and quantified for the determination of the entrapment efficiency of both liposomes and Et-PEVs. Similar values of entrapment efficiency were found, being higher than 85% for both kaempferol derivatives (Table 5). This proves the loading capabilities of the developed vesicles, regardless of the presence of ethanol. Landi-Librandi et al. incorporated kaempferol and other flavonoids into soy phosphatidylcholine liposomes, which showed fluctuations in the entrapment efficiency of kaempferol as a function of the use of cholesterol or cholesteryl ethyl ether, resulting in values of 45–60% and 77–87%, respectively [43].

**Figure 2:**
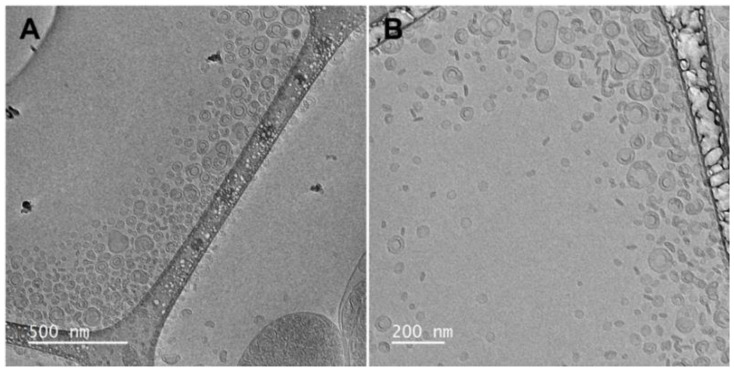
Cryo-TEM micrographs of horseradish extract vesicles: 15,000× (**A**) and 20,000× (**B**) magnifications are displayed.

The vesicles’ morphology was investigated via cryo-TEM observation. The micrographs showed the co-existence of spherical bilamellar vesicles and elongated unilamellar vesicles (Figure 2) below 100 nm in diameter, in alignment with the light scattering data (Table 4). The combination of the two techniques represents a reliable strategy to describe the vesicles’ formation and morphology [44].

**Figure 3:**
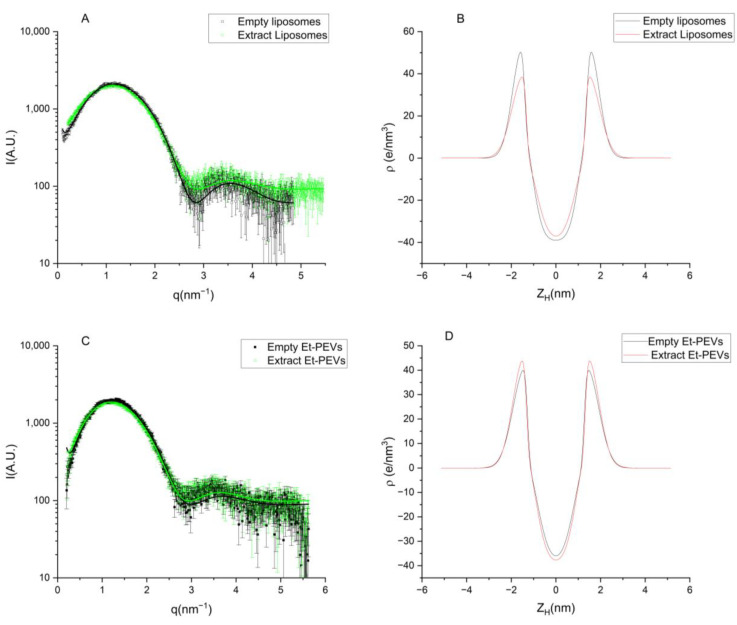
SAXS profiles of liposomes (**A**) and Et-PEVs (**C**). The lines correspond with the fits to Gaussian lamellar model. Electron density profiles of liposomes (**B**) and Et-PEVs (**D**).

The structure of the vesicles was further studied via SAXS patterns. Figure 3 shows the SAXS curves and the fits to the lamellar model, which are distinctive to bilayered structures. More specifically, the profiles suggest that both liposomes and Et-PEVs had a predominant unilamellar arrangement (*N* = 1; Table 6). The presence of some bilamellar structures, as observed in the microscopy study, is not incompatible with the unilamellar observation in SAXS: because of the large distances (10–20 nm) and corresponding low lamellar correlation, the correlation peak is not likely to be observed.

The parameters that describe the bilayer were obtained from the fits to the lamellar model and are listed in Table 6. *Z_H_*, the distance between the polar head and the center of the bilayer, decreased slightly as the extract was loaded into liposomes (14.2 Å) as compared to empty liposomes (15.0 Å). The opposite behavior was found in Et-PEVs: the loading of the extract increased *Z_H_* (14.2 Å) in comparison with empty Et-PEVs (13.7 Å). Moreover, the effect of ethanol was markedly visible, as *Z_H_* decreased from 15.0 Å in empty liposomes to 13.7 Å in empty Et-PEVs. These findings correspond well to the decrease in liposomes’ size and the enlargement of Et-PEVs detected via light scattering measurements when the extract was incorporated (Table 4). Similarly, the decrease in *Z_H_* due to ethanol reflects the decrease in size detected in empty Et-PEVs vs. empty liposomes. Conversely, *σ_H_*, the polar head amplitude, increased in liposomes and decreased in Et-PEVs upon incorporation of the extract.

These variations are reasonably due to the interactions of the extract with ethanol and the phospholipids, which arrange differently to accommodate the diverse components of the extract.

### 3.3. Biocompatibility and Antioxidant Activity

The absence of hemolytic activity is one of the most common indicators of the biocompatibility of nanoparticles [45]. In this study, no erythrocyte-disrupting ability was found for the horseradish extract at low concentrations (below 1 mg/mL). The incubation with the extract solution at 1 mg/mL produced a hemolytic activity of 1.9%, which decreased significantly to 0.9% when the extract was formulated in liposomes and to ca. 1.2% in Et-PEVs without statistical significance (Table 7). As expected, the nanocarriers were not harmful to erythrocytes: the hemolytic activity of the empty vesicles was low and similar to that of the corresponding extract-loaded vesicles (Table 7).

The incubation with the extract solution at 2 mg/mL produced a marked increase in the hemolytic activity to ca. 16%, which is way above the 5% threshold that is considered to be of no appreciable risk to erythrocytes [46,47]. However, hemolysis decreased dramatically when the extract was incorporated into the vesicles (1.5% in liposomes and 3.2% in Et-PEVs), pointing to a strong protective effect of the nanocarriers. The empty vesicles confirmed their non-toxicity at this concentration, as well. As a matter of fact, colloidal carriers—particularly liposomes and deformable lipid vesicles—are often used to reduce the hemolytic activity of active compounds, providing at the same time a safe delivery and increased drug penetration [48,49].

**Figure 4:**
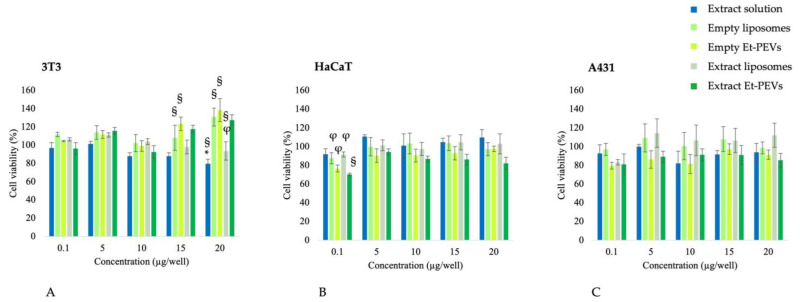
Cell viability ((**A**) 3T3; (**B**) HaCaT; (**C**) A431) upon 24 h exposure to horseradish extract formulations. Values are the means ± standard error; *n* = 2 independent experiments; ^§^ *p* < 0.05 vs. control untreated cells (100% viability); * *p* < 0.01 vs. extract liposomes; ^φ^ *p* < 0.05 vs. extract Et-PEVs.

The biocompatibility of the nanoformulations was also studied in skin cells (3T3, HaCaT, A431). Cell viability results are presented in Figure 4. In 3T3 cells, a slight reduction in viability was induced by the extract solution, yet never lower than 80% (Figure 4A). The empty vesicles did not show cytotoxicity but rather a slight proliferation (*p* < 0.05 vs. control untreated cells; Figure 4A), likely due to the components of S75 used for their preparation (e.g., phosphatidylcholine, phosphatidylethanolamine, lysophosphatidylcholine, triglycerides, free fatty acids, and alpha-tocopherol). Thanks to this effect, the liposomes prevented the mild cytotoxicity induced by the extract solution, giving viability values similar to those of the control untreated cells (100% viability). The same behavior was observed for Et-PEVs, which, at the higher concentrations, protected the cells but did not induce cell proliferation (Figure 4A).

In HaCaT cells, no particular cytotoxicity emerged. Et-PEVs decreased cell viability slightly, ranging from 95% to 70% as a function of the extract concentration (Figure 4B). Similarly, in A431 cells, no relevant cytotoxicity was detected (Figure 4C). Cell viability values were always above 80%, with no statistical differences among treatments. In alignment with our findings, the retention of cell viability by the horseradish extract has been proven in several studies using different cell lines [3,6,50,51].

In order to further study the applicability of the prepared horseradish nanoformulations, their antioxidant activity was estimated as radical scavenging and ferric-reducing abilities. In vitro colorimetric assays are widely used to predict the ability of free or nanoformulated active compounds to prevent the harmful effects of free radical species in the human body [52,53,54,55]. The extract solution scavenged the DPPH radical moderately (40%, corresponding to ~80 μg/mL of Trolox equivalents; Table 8). The antioxidant activity doubled (80%; Table 8) when the extract was formulated in the vesicles, thanks to a contribution from the phospholipids of the nanocarriers, as demonstrated by the antioxidant activity of the empty vesicles (60%; Table 8). Similarly, the ferric-reducing ability of the extract was potentiated by the nanoformulation since the values increased from ca. 730 to 1100 μg/mL of ferrous equivalents (Table 8).

## 4. Conclusions

A horseradish root extract was formulated in phospholipid vesicles to overcome common problems of natural extracts, such as poor stability and bioavailability, and to potentially enhance biological activity. The prepared conventional liposomes and Et-PEVs showed a nanometric size and high entrapment efficiency of two flavonoids identified in the extract. According to the results from cell studies, the vesicles were cytocompatible. No relevant alterations in the viability of keratinocytes and fibroblasts were detected. Noteworthily, the nanoformulations decreased the hemolytic activity of the extract significantly. Additionally, the antioxidant activity of the extract was potentiated when formulated in liposomes and Et-PEVs, thanks to a contribution from the nanocarriers’ phospholipids. In light of the overall findings, the developed formulations offer a viable method of delivering the investigated extract to the skin for a possible application against oxidative stress-correlated disorders. Further research—such as in vivo testing—should be conducted to substantiate the in vitro results and promote a plant-based nanomedicine approach.

## Figures and Tables

**Table 1:** Composition of the nanoformulations.

Nanoformulation	S75	Horseradish Extract	Et	Water
**Horseradish extract liposomes**	180 mg	20 mg		1 mL
**Empty liposomes**	180 mg			1 mL
**Horseradish extract Et-PEVs**	180 mg	20 mg	0.1 mL	0.9 mL
**Empty Et-PEVs**	180 mg		0.1 mL	0.9 mL

S75, phospholipids; Et, ethanol 96%.

**Table 3:** Concentrations of target compounds of the horseradish extract (mg/g of dried extract mass (dm); means ± SD, *n* = 3).

Compound	Peak No. ^§^	Horseradish Extract (mg/g dm)
**Sulfur and nitrogen compounds (glucosinolates)**		**0.44 ± 0.03**
2-Hydroxypropyl glucosinolate (sinigrin)	**1**	0.31 ± 0.02
Butyl and/or methylpropyl glucosinolate (glucocochlearine and/or glucoconringianine) ^a^	**5**	0.01 ± 0.00
3-Indolylmethyl glucosinolate (glucobrassicin)	**9**	0.05 ± 0.00
2-Phenethyl glucosinolate (gluconasturtiin) ^b^	**11**	0.07 ± 0.01
**Nitrogen compounds**		**1.49 ± 0.13**
Uridine	**2**	0.24 ± 0.01
Tyrosine	**3**	0.55 ± 0.05
Phenylalanine	**4**	0.65 ± 0.07
Tryptophan	**8**	0.05 ± 0.00
**Hydroxybenzoic and phenyl derivatives**		**1.15 ± 0.09**
Dihydroxybenzoic acid glucoside I ^c^	**6**	0.43 ± 0.03
Dihydroxybenzoic acid glucoside II ^c^	**7**	0.37 ± 0.02
Dihydroxybenzoic acid ^c^	**10**	0.26 ± 0.02
Vanilloyl exoside ^c^	**13**	0.03 ± 0.00
Phenethyl rutinoside ^d^	**14**	0.02 ± 0.00
Sinapinic acid	**15**	0.03 ± 0.01
**Total Flavonols**		**0.16 ± 0.01**
Kaempferol 3-*O*-rutinoside	**16**	0.11 ± 0.01
Kaempferol dipentoside ^e^	**18**	0.05 ± 0.00

^§^ peak number as reported in Table 2; ^a^ expressed as sinigrin equivalents; ^b^ expressed as glucobrassicin; ^c^ expressed as vanillic acid equivalents; ^d^ expressed as tyrosol equivalents; ^e^ expressed as kaempferol 3-*O*-rutinoside equivalents.

**Table 4:** Characteristics of the nanoformulations.

	Empty Liposomes	Empty Et-PEVs	Horseradish Extract Liposomes	Horseradish Extract Et-PEVs
Mean diameter (nm)	96 ± 4.2	8 ± 5.6 *	84 ± 5.3 °	96 ± 6.9 ^§,^#
Polydispersity index	0.34 ± 0.05	0.51 ± 0.07 *	0.22 ± 0.02 °	0.23 ± 0.02 ^§^
Zeta potential (mV)	−69 ± 8.4	−73 ± 5.2	−52 ± 3.6 °	−63 ± 4.9 ^§,^#

Values are the means ± SD (*n* > 10); * statistically different (*p* < 0.001) from empty liposomes; ° statistically different (*p* < 0.001) from empty liposomes; § statistically different (*p* < 0.001) from empty Et-PEVs; # statistically different (*p* < 0.001) from extract liposomes.

**Table 5:** Entrapment Efficiencies (EEs) of the flavonoids identified in the horseradish extract. Values are the means ± SD (*n* = 4).

Peak No. ^§^	Compound	EE (% ± SD)	
		Horseradish Extract Liposomes	Horseradish Extract Et-PEVs
**16**	Kaempferol-3-*O*-rutinoside	86 ± 3.8	88 ± 4.1
**18**	Kaempferol di-pentoside ^a^	88 ± 8.4	87 ± 7.4

^§^ peak number as reported in Table 2; ^a^ expressed as kaempferol-3-*O*-rutinoside equivalents.

**Table 6:** Fitting and derived parameters from the SAXS curves of the nanoformulations. Values are the means ± standard deviations. *χ*^2^: reduced chi squared; *N*: number of correlated bilayers; *Z_H_*: polar head Gaussian center; *σ_H_*: polar head Gaussian amplitude.

	Empty Liposomes	Empty Et-PEVs	Horseradish Extract Liposomes	Horseradish Extract Et-PEVs
***χ* ^2^**	1.2	1.9	1.0	1.6
***N***	1.0	1.0	1.0	1.0
***Z_H_*** (***Å***)	15.0 ± 0.5	13.7 ± 0.5	14.2 ± 0.5	14.2 ± 0.5
***σ_H_*** (***Å***)	4.44 ± 0.5	5.41 ± 0.5	5.33 ± 0.5	5.00 ± 0.5

**Table 7:** Hemolytic activity of horseradish extract nanoformulations.

Formulation	Hemolysis (%)	
	1 mg/mL	2 mg/mL
**Horseradish extract solution**	1.9 ± 0.18	16.3 ± 2.42
**Empty liposomes**	0.9 ± 0.60	** 1.8 ± 0.77
**Empty Et-PEVs**	* 1.3 ± 0.10	** 1.1 ± 0.02
**Horseradish extract liposomes**	* 0.9 ± 0.02	** 1.5 ± 0.44
**Horseradish extract Et-PEVs**	1.2 ± 0.45	** 3.2 ± 0.97

Values are the means ± standard deviations (*n* = 3); * *p* < 0.05 vs. 1 mg/mL extract solution; ** *p* < 0.01 vs. 2 mg/mL extract solution.

**Table 8:** Antioxidant activity of the horseradish formulations. Results of the DPPH assay are expressed as AA (%) and TE (μg Trolox equivalents/mL); results of the FRAP assay are expressed as FE (µg ferrous equivalents/mL). Values are the means ± standard deviations of three separate experiments, each performed in quadruplicate. * *p* < 0.05 vs. extract solution.

Formulation	DPPH Assay		FRAP Assay
	AA (%)	TE (µg Trolox Equivalents/mL)	FE (µg Fe^2+^ Equivalents/mL)
**Horseradish extract solution**	40 ± 6.7	81 ± 4.9	733 ± 6.8
**Empty liposomes**	60 ± 8.5	121 ± 13.5	687 ± 32.4
**Empty Et-PEVs**	62 ± 0.3	133 ± 5.4	637 ± 26.7
**Horseradish extract liposomes**	* 80 ± 8.3	* 167 ± 19.7	* 1090 ± 9.1
**Horseradish extract Et-PEVs**	* 81 ± 7.2	* 168 ± 11.6	* 1180 ± 37.5

## Data Availability

The analyzed data sets generated during the present study are available from the corresponding author upon reasonable request.
